# Effectiveness of lithium in subjects with treatment-resistant depression and suicide risk: results and lessons of an underpowered randomised clinical trial

**DOI:** 10.1186/1756-0500-7-731

**Published:** 2014-10-17

**Authors:** Francesca Girlanda, Andrea Cipriani, Emilia Agrimi, Maria Grazia Appino, Andrea Barichello, Rossella Beneduce, Irene Bighelli, Giulia Bisoffi, Alfredo Bisogno, Paola Bortolaso, Marianna Boso, Carmela Calandra, Liliana Cascone, Mariasole Castellazzi, Caterina Corbascio, Vincenzo Fricchione Parise, Francesco Gardellin, Daniele Gennaro, Batul Hanife, Camilla Lintas, Marina Lorusso, Antonina Luca, Maria Luca, Chiara Luchetta, Claudio Lucii, Francesca Maio, Alessandra Marsilio, Chiara Mattei, Daniele Moretti, Michela Nosè, Guglielmo Occhionero, Duccio Papanti, Damiano Pecile, Mauro Percudani, Davide Prestia, Marianna Purgato, Francesco Restaino, Salvatore Romeo, Tiziana Sciarma, Stefania Strizzolo, Stefania Tamborini, Orlando Todarello, Fiorella Tozzi, Simona Ziero, Spyridon Zotos, Corrado Barbui

**Affiliations:** Dipartimento di Sanità Pubblica e Medicina di Comunità, Sezione di Psichiatria, Università di Verona, Verona, Italy; Department of Psychiatry, Warneford Hospital, Oxford, UK; Servizio Psichiatrico di Diagnosi e Cura, Istituti Ospitalieri di Cremona, Cremona, Italy; Dipartimento di Salute Mentale, Centro di Salute Mentale di Finale Ligure, Asl n°2, Savona, Italy; IRCCS “Centro San Giovanni di Dio” FBF, Brescia, Italy; Ufficio Supporto alla Ricerca e Biostatistica, Azienda Ospedaliera Universitaria Integrata di Verona, Verona, Italy; Dipartimento di Salute Mentale, UO Salute Mentale Cava de’Tirreni - Costa d’Amalfi, ASL, Salerno, Italy; Servizio Psichiatrico di Diagnosi e Cura Cittiglio, Psichiatria del presidio del Verbano, Ospedale di Circolo e Fondazione Macchi, Varese, Italy; Azienda Ospedaliera di Pavia e Dipartimento di Neuroscienze, Centro Psico-Sociale di Pavia, Sezione di Psichiatra, Università di Pavia, Pavia, Italy; Dipartimento di Specialità Medico-Chirurgiche, Sezione di Psichiatria, Azienda Ospedaliero Universitaria, “Policlinico-Vittorio Emanuele”, Catania, Italy; Dipartimento di Salute Mentale, Asl AT, Asti, Italy; Asl Avellino (Regione Campania), U.O.C. di Salute Mentale di Avellino, Avellino, Italy; Dipartimento di Salute Mentale, Ulss 6, Vicenza, Italy; Azienda Ospedaliera SS Antonio e Biagio, Alessandria, Italy; 1° Servizio autonomo di Psichiatria, Ulss 20, Verona, Italy; Università “Aldo Moro” Bari, Bari, Italy; Dipartimento GF Ingrassia, Sezione di Neuroscienze, Azienda Ospedaliero Universitaria “Policlinico-Vittorio Emanuele” Catania, Catania, Italy; Dipartimento di Salute Mentale, Azienda per i Servizi Sanitari n°1 Triestina, Trieste, Regione FVG, Italy; Dipartimento Interaziendale di Salute Mentale e Dipendenze di Siena, Siena, Italy; Dipartimento di Salute Mentale, Ulss 18, Rovigo, Italy; Clinica Psichiatrica dell’Università di Genova, Genova, Italy; S.O.C Psichiatria Asti Centro-Nord, Asl AT, Asti, Italy; Unità Operativa di Psichiatria n° 62, Bollate, DSM A.O. “G. Salvini”, Garbagnate Milanese, Italy; Dipartimento di Medicina Sezione di Psichiatria Psicologia Clinica e Riabilitazione Psichiatrica, Università di Perugia, Perugia, Italy; Azienda Uls di Ferrara, Ferrara, Italy

**Keywords:** Randomised controlled trial, Lithium, Suicide, Deliberate self harm, Mortality, Treatment resistant depression

## Abstract

**Background:**

As lithium treatment might be effective in reducing the risk of deliberate self-harm (DSH) in adult patients with unipolar affective disorders, we designed a pragmatic randomised trial to assess its efficacy in more than 200 patients with treatment-resistant depression. However, we randomised 56 patients only. The aim of this report is therefore twofold: first, to disseminate the results of this underpowered study which may be incorporated into future meta-analytical reviews; second, to analyse some critical aspects of the study which might explain failure to reach the target sample size.

**Methods:**

We carried out a randomised, parallel group, assessor-blinded superiority clinical trial. Adults with a diagnosis of major depression, an episode of DSH in the previous 12 months and inadequate response to at least two antidepressants given sequentially at an adequate dose for an adequate time for the current depressive episode were allocated to add lithium to usual care (intervention arm) versus usual care alone (control arm). Suicide completion and acts of DSH during the 12 months of follow-up constituted the composite primary outcome.

**Results:**

Of 58 patients screened for inclusion, 29 were allocated to lithium plus usual care and 27 were assigned to usual care without lithium. Six patients in the lithium plus usual care group and seven in the usual care group committed acts of DSH during the follow-up phase. The survival probability did not differ between the two treatment arms (Chi^2^ = 0.17, p =0.676). With regard to changes in the severity of depressive symptomatology from baseline to endpoint, no significant differences were detected.

**Conclusions:**

The present study failed to achieve the minimum sample size needed to detect a clinically meaningful difference between the two treatment arms. Consequently, the finding that lithium, in addition to usual care, did not exert a positive effect in terms of reduction of DSH after 12 months of follow-up is likely due to the lack of sufficient statistical power to detect a difference, if a difference existed. The dissemination of the results of this underpowered study will inform future meta-analytical reviews on lithium and suicide-related outcomes.

**Trial registration:**

ClinicalTrials.gov identifier: NCT00927550

## Background

There is some evidence that lithium treatment might be effective in reducing the risk of suicide-related outcomes in patients with treatment-resistant depression (TRD) [1–3]. A recent systematic review of 48 randomized trials (6674 participants), which investigated the effect of lithium on the risk of suicide and deliberate self-harm (DSH) in patients with mood disorders, found that lithium was more effective than placebo in reducing the number of suicides (odds ratio (OR) 0.13, 95% confidence interval (CI) 0.03 to 0.66) and deaths from any cause (OR 0.38, 95% CI 0.15 to 0.95) [4]. However, no clear benefits were observed for lithium compared with placebo in preventing deliberate self-harm (OR 0.60, 95% CI 0.27 to 1.32) [4]. In unipolar depression, lithium was associated with a reduced risk of suicide (OR 0.36, 95% CI 0.13 to 0.98), although the confidence interval around the point estimate ranged from substantial beneficial effect to almost no effect. Of note, the included trials were not primarily designed to measure this outcome, and heterogeneous patient populations were enrolled. Additionally, while some trials included acutely depressed patients, euthymic cases were enrolled in other studies.

On these grounds, we designed a pragmatic randomised trial to assess whether adding lithium to usual care is an effective treatment strategy to reduce the risk of suicidal behaviour in long term treatment of people with TRD and previous history of DSH. As reported in the study protocol [5], we initially planned to include more than 200 participants, but we managed to randomise 56 patients only. The aim of this report is therefore twofold: first, to disseminate the results of this underpowered study which may be incorporated into future meta-analytical reviews; second, to analyse some critical aspects of the study which might explain failure to reach the target sample size.

## Methods

### Study design

The study was funded by the Italian Medicine Agency and received ethical approval in each participating site. The Ethics Committee in Verona (coordinating centre) approved the study on May 6th 2009 (Study code: LAST-RD - FARM77Z3BL5.101-03-2009; Approval number 1675). A detailed description of the study methods has already been published [5]. Patients with a diagnosis of TRD and history of DSH were randomly allocated to (i) add lithium to usual care or (ii) usual care without lithium. Patients were randomly assigned to one of the two treatment groups with an equal probability of assignment to each treatment (allocation ratio 1:1). A centralised randomisation procedure was employed to preserve allocation concealment and stratified by presence or absence of Axis II diagnosis. In order to resemble ordinary practice, patients and clinicians were not blind to treatments provided during the trial. However, to limit the potential for ascertainment bias, an independent adjudicating committee, blind to treatment allocation, validated the events that constituted the primary outcome. Patients were assessed at baseline and then every month after random allocation until the completion of the 12-month follow-up, using an ad hoc and structured form. Acts of DSH and suicide during the 12 months of follow-up constituted the composite primary outcome. A sample size calculation [5] revealed that 210 patients would have 80% power to show a clinically significant advantage associated with lithium treatment. All phases of the trial were recorded following the CONSORT statement [6, 7].

### Inclusion criteria

The following inclusion criteria were adopted: (a) diagnosis of unipolar major depression (clinical diagnosis, guided by DSM-IV criteria); (b) an episode of DSH in the previous 12 months; (c) inadequate response to at least two antidepressants given sequentially at an adequate dose for an adequate time for the current depressive episode; (d) uncertainty about which treatment arm would be best for the participant; (e) age 18 or above; (f) to sign written informed consent.

### Exclusion criteria

The following exclusion criteria were adopted: (a) a primary diagnosis of any concurrent Axis I disorder (according to DSM-IV criteria) other than major depression (by contrast, any concurrent DSM-IV Axis II disorder was not an exclusion criterion); (b) previous exposure to lithium associated with lack of efficacy or adverse reactions; (c) clinical conditions contraindicating lithium (i.e., thyroid or kidney disease/abnormalities); (d) pregnant/lactating women and women of childbearing potential not practicing a reliable method of contraception.

### Assessments

The following information was collected at baseline: socio-demographic and clinical characteristics, diagnosis according to the Mini Neuropsychiatry Interview (MINI) [8], severity of illness according to the Quick Inventory Depression Scale (QIDS) [9], a self-rated instruments that has been shown to have good psychometric properties [10]. Follow-up data were obtained monthly after random allocation using an electronic form, as follows: any death or DSH, lithium oral dose and plasma level (if applicable), and QIDS scores. Patients allocated to lithium were administered an oral starting dose ranging between 150 and 300 milligrams (according to clinical judgement). Suggested final oral dose had to achieve plasma levels from 0.4 to 1.0 mmol/L. Clinicians were free of increasing or decreasing the dose according to clinical status and circumstances.

### Statistical analysis

All randomized subjects with at least one post-baseline assessment were considered in the analysis of primary and secondary outcomes. Patients with missing values and lost during follow-up contributed to the analysis of the primary and secondary outcomes only for the time during which data are available (censoring). Missing values in depressive symptom ratings were imputed using the last observation carried forward (LOCF) approach: depressive ratings were carried forward from the last available assessment to the 12-month follow up assessment. Additionally, patients in each arm were always analysed according to the corresponding treatment group’s allocation at baseline.

Kaplan-Meier estimates for the time from randomised treatment assignment until the first event that constitutes the primary outcome were plotted to compare the treatment’s effect, and log-rank test was performed to test for differences.

Change in severity of depressive symptoms from baseline to 12 months was compared between the two groups of treatment through appropriate statistical methods for repeated measurements (paired t-test or McNemar non parametric test according to the variables distribution).

## Results

### Socio-demographic and clinical characteristics

**Figure 1:**
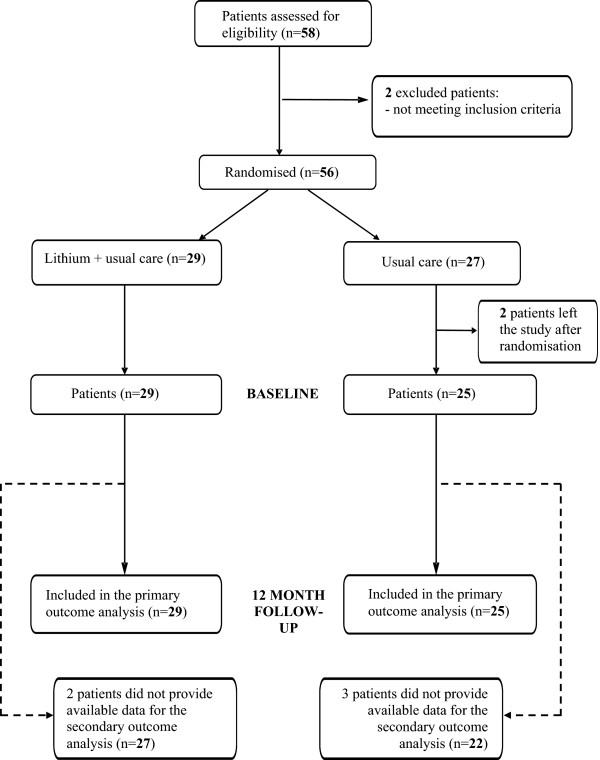
**Study flow-diagram.**

Of 58 patients screened for inclusion, a total of 56 were enrolled in the study and randomly assigned to treatment (Figure 1); 29 subjects were allocated to lithium plus usual care and 27 were assigned to usual care without lithium. Two patients withdrew consent and left the study immediately after random allocation. Five additional patients withdrew from the study during follow-up (Figure 1). Table 1 shows the baseline socio-demographic and clinical characteristics of the study sample. The majority of study participants were female, married, with high level of education; the mean age was 46 and 47 years in the lithium and usual care group, respectively.

**Table 1:** **Distribution of patients’ socio-demographic and clinical characteristics by allocated treatment**

	Lithium + Usual care (n = 29)		Usual care (n = 25)		p
	N	%/mean	n	%/mean	
Females (%)	16	55	18	72	0.202
Age (years), mean (SD)	29	46 (12.3)	25	47 (9.4)	0.853
Education (%)					0.951
Primary school certificate	13	45	11	44	
High school education/degree	16	55	14	56	
Occupational status (%)					0.342
Employed	15	52	8	32	
Unemployed	6	21	7	28	
Other	8	28	10	40	
Marital status					0.944
Unmarried	8	28	6	24	
Married	15	52	14	56	
Divorced/Widowed	6	21	5	20	
Psychiatric admissions in the previous 12 months (%)					
0	10	34	7	28	
1	11	38	9	36	0.781
≥2	8	28	9	36	
Personality disorders (%)	10	34	7	28	0.609
Current alcohol/drug abuse	0	0	2	8	0.210
History of alcohol abuse	4	14	8	32	0.188
Acts of self-harm in the previous 12 months (%)					0.721
1	16	55	15	60	
≥2	13	45	10	40	
Physical illness (%)	12	41	5	20	0.092
Past use of antidepressants (number), mean (SD)	29	3 (1.9)	25	3 (1.9)	0.470
Past use of lithium (%)	4	14	3	12	1.000

Legend: *SD* = Standard deviation.

The majority of patients were admitted to a psychiatric facility in the previous 12 months, one third had a concurrent diagnosis of personality disorder, and only a minority suffered from alcohol or drug use problems. A proportion of 45% in the lithium group and 40% in the usual care group had two or more acts of DSH recorded in the previous 12 months. Only a minority of cases had already been exposed to lithium in the past (Table 1).

Table 2 presents the use of antidepressants, antipsychotics, benzodiazepines and mood stabilizers during the study period. Patients allocated to lithium used less often antipsychotics and mood stabilizers, although differences were not statistically significant.

**Table 2:** **Use of antidepressants, antipsychotics, benzodiazepines and mood stabilizers during the study period**

	Lithium + Usual care (n = 29)		Usual care (n = 25)		p
	N	%	n	%	
Antidepressants	26	96	23	100	1.000
Antipsychotics	16	59	16	70	0.449
Benzodiazepines	23	85	20	87	0.100
Mood stabilizers	10	37	10	43	0.643

### Lithium efficacy

Lithium was administered during the study at a mean dose of 444 mg (Standard Deviation (SD) =304), with a mean blood level of 0.57 mEq/L (SD =0.24). A total of 11 patients allocated to lithium experienced adverse effects that the treating psychiatrist attributed to lithium.

**Figure 2:**
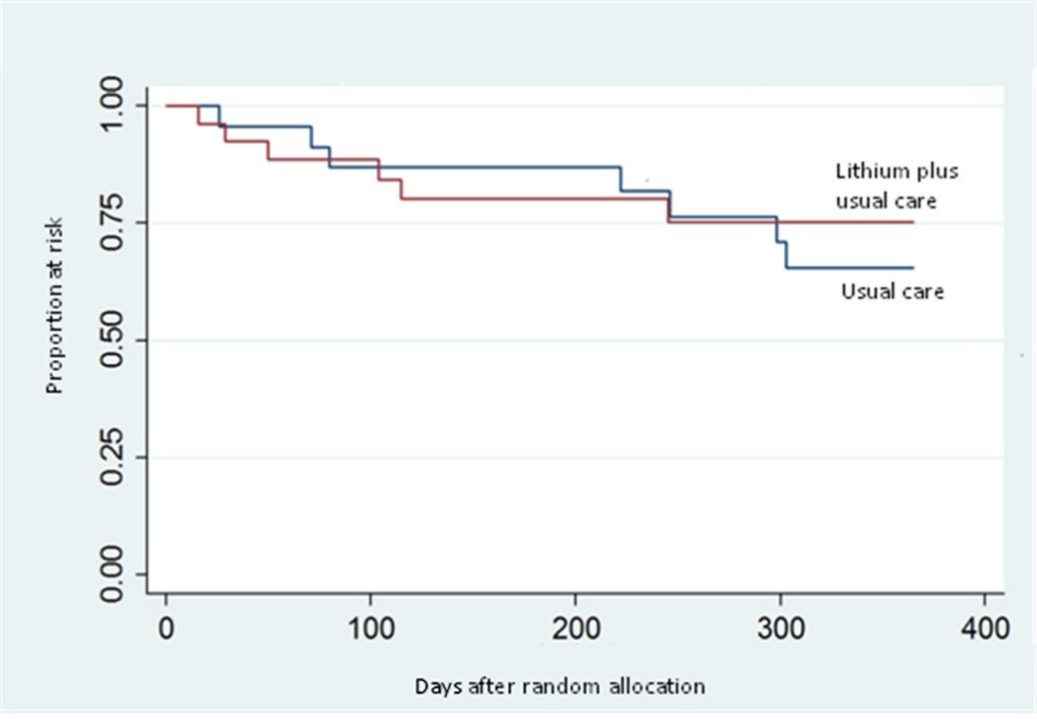
**Kaplan-Meier curve showing the survival probability in patients allocated to lithium plus usual care (n = 29) versus usual care (n = 27).** Acts of deliberate self-harm were the outcome measure.

Six patients in the lithium plus usual care group and seven in the usual care group committed acts of DSH during the follow-up phase; one of the six patients in the lithium plus usual care group died by suicide versus none of the seven patients in the usual care group. Figure 2 shows the Kaplan-Meier survival curve for the primary outcome. The survival probability did not differ between the two treatment arms (Chi^2^ = 0.17, p =0.676). The survival probability at 12 month was 75% and 65% in the lithium and standard therapy group, respectively.

With regard to changes in the severity of depressive symptomatology from baseline to endpoint, no significant differences were detected (Table 3).

**Table 3:** **Mean change in depressive symptomatology from baseline to follow-up**

	Lithium + Usual care (n = 27)	Usual care (n = 22)	P
QIDS at baseline, mean (SD)	16.81 (4.05)	20.05 (2.89)	
Mean change (SD) [confidence interval]	−7 (8.2) [−10.2, −3.80]	−6.1 (5.7) [−8.62, −3.56]	0.951

Legend: *SD* = Standard deviation.

## Discussion

The present study failed to achieve the minimum sample size needed to detect a clinically meaningful difference between the two treatment arms. Consequently, the finding that lithium, in addition to usual care, did not exert a positive effect in terms of reduction of DSH after 12 months of follow-up is likely due to the lack of sufficient statistical power to detect a difference, if a difference existed (type-II error). Considering that most individual studies carried out on this topic suffer from low statistical power [11], it is likely that only systematic reviews of clinical trial data, by pooling together all available studies, will have a chance of detecting clinically meaningful differences. It is therefore of paramount relevance that the results of all randomised evidence is published and available to researchers, irrespective of statistical power or study outcome.

A second relevant aspect is to analyse the critical features of the study which might explain failure to reach the target sample size, in order to assist investigators designing future studies on this topic. First, this study was aimed at providing a scientific answer to a clinically relevant question enrolling real-world patients. We were able to build a research network of mental health professionals who accepted the idea of using their everyday clinical practice to produce scientific knowledge. This research infrastructure proved to work efficiently in a previous experimental study which was conducted and finalised effectively [12, 13]. On this occasion, however, of more than 50 Italian community psychiatric services that were initially interested in the study, only 22 were able to contribute and randomised at least one patient. Reasons for not contributing included lack of staff that might follow the process of ethics committee approval or, for some services where the protocol was approved, lack of dedicated personnel to actually recruit patients. The availability of study funding provided by the Italian Medicine Agency failed to overcome these practical issues, as funding was allocated to each service too late, due to a very long and time-consuming administrative process.

Second, despite an attempt was made to define broad and clinically-sound entry criteria, very few eligible cases were identified (Figure 1). The main issue was that most patients seen in clinical practice with a recent history of DSH and a depressive episode did not have a diagnosis of unipolar depression but, rather, of bipolar depression. In our study, however, a diagnosis of bipolar depression was an exclusion criterion, as data have already shown a beneficial effect of lithium in these patients. In addition, in recent years there is a trend of interpreting patients with affective features and acts of DSH as having bipolar disorder more than unipolar depression, so that even patients with a history of depressive episodes and some acts of DSH were considered cases belonging to the so-called bipolar spectrum, being therefore ineligible for the study [14].

Third, some patients were not considered eligible because the treating psychiatrists deemed as not clinically reasonable the use of lithium. The main concern was the use of a medicine with a narrow therapeutic/toxic ratio, which requires regular serum level tests. Lithium overdose may cause nausea, emesis, diarrhea, asthenia, ataxia, confusion, lethargy, polyuria, seizures and coma. Other toxic effects of lithium include coarse tremor, muscle twitching, convulsions and renal failure. People who survive a poisoning episode may develop persistent neurotoxicity [15]. Clearly, in patients selected on the basis of a recent episode of self-harm, doctors were reluctant to prescribe a potentially dangerous mean which could be used for committing other acts of DSH. The counterargument of the evidence base suggesting a potential beneficial rather than harmful effect of lithium in the long-term was not convincing when individual cases were approached.

Fourth, in patients with TRD lithium is not the only therapeutic options, as second-generation antipsychotics (SGAs) have been increasingly tested in clinical trials and used in clinical practice [16]. A recent systematic review identified 14 randomised trials of aripiprazole, olanzapine/fluoxetine combination, quetiapine, and risperidone [17]. It found that SGAs for the adjunctive treatment of depression are efficacious in reducing observer-rated depressive symptoms, although the benefit is small to moderate and short-term. Quetiapine in several countries, including Italy, has a licensed indication for the pharmacological treatment of TRD. On clinical grounds, therefore, the addition of one of the SGAs may be considered more suitable and less dangerous option than the addition of lithium. SGAs, additionally, do not require that strict monitoring that must be followed with lithium treatment. These considerations may have prevented doctors to include several potentially eligible patients into the trial.

## Conclusions

The dissemination of the results of this underpowered study may inform future meta-analytical reviews on lithium and suicide-related outcomes. As the problem of missing trials is one of the greatest ethical and practical problems facing medicine today, it is considered imperative that all trials are registered and their results fully published [18].
